# Matrix Metalloproteinase‐13 Is an Unfavorable Prognostic Factor in Chordoma by Digesting Growth Inhibitory Collagens

**DOI:** 10.1002/cam4.71662

**Published:** 2026-02-19

**Authors:** Yumiko Oishi, Katsuhiro Kawaai, Ryota Tamura, Yukiko Kuroda, Shinobu Noji, Masahiro Toda, Koichi Matsuo

**Affiliations:** ^1^ Department of Neurosurgery Keio University School of Medicine Tokyo Japan; ^2^ Laboratory of Cell and Tissue Biology Keio University School of Medicine Tokyo Japan

**Keywords:** chordoma, MMP13, safranin‐O staining, type II collagen

## Abstract

**Objective:**

Chordomas are low‐grade but invasive tumors with limited therapies. Some conventional chordomas exhibit cartilage‐like extracellular matrix (ECM). This study examined the expression and clinical significance of collagenases in chordomas.

**Methods:**

Brachyury‐positive primary chordoma tissues were analyzed by immunohistochemistry for matrix metalloproteinase (MMP)13, MMP9, and cathepsin K, and by immunofluorescence for MMP13 and MMP9. ECM phenotype was categorized using safranin‐O staining, where safranin‐O marks cartilage‐like ECM. We compared MMP13 expression scores and progression‐free survival (PFS) between safranin‐O‐negative and ‐positive groups. Collagenase gene expression and protein localization in JHC7 cells were analyzed by quantitative PCR and immunocytochemistry, respectively. Collagen digestion activity was evaluated using fluorescein isothiocyanate–labeled type II collagen (COL2) in the presence or absence of an MMP13‐specific inhibitor. Cell growth was evaluated in the presence of type I collagen (COL1) or COL2.

**Results:**

Safranin‐O negative chordomas had shorter PFS than safranin‐O positive chordomas (*p* = 0.016). MMP13 was expressed in human chordoma tissues and JHC7 cells; the MMP13 expression score was higher in safranin‐O‐negative chordomas than in safranin‐O‐positive chordomas (*p* = 0.018). JHC7 cells digested COL2, and digestion was partially but significantly inhibited by an MMP13‐specific inhibitor (*p* < 0.05). COL2 inhibited the growth of JHC7 cells more strongly than COL1 in a dose‐dependent manner (*p* < 0.01).

**Conclusions:**

MMP13 may promote aggressive behavior in chordoma by degrading growth‐inhibitory COL2‐rich ECM. These data support MMP13 as a potential unfavorable prognostic marker and therapeutic target in chordoma.

## 1 Introduction

Chordomas are slow‐growing but locally invasive and clinically aggressive tumors, with phenotypes that recapitulate notochordal differentiation in the axial skeleton [1, 2]. Chordoma cells express the transcription factor brachyury, which is known to be an essential regulator of notochord development, and this expression serves to confirm the diagnosis [3]. The tumors are generally located in the skull base (32%), mobile spine (32.8%), and sacrum (29.2%) [4]. Patients with skull base chordomas develop severe symptoms, including cranial nerve palsies and brain stem compression, making surgical treatment challenging. Furthermore, chordomas are unresponsive to chemotherapy or radiotherapy [1, 2], and palliative tumor debulking followed by radiation therapy is thus the standard treatment; however, therapy is often not curative [1, 2, 5]. The median survival of patients with chordoma is 7.7 years, and its prognosis is poor, with a 5‐year survival rate of 72%, decreasing to 48% and 31% at 10 and 20 years, respectively [6].

The current World Health Organization (WHO) classification divides chordomas into conventional, dedifferentiated, and poorly differentiated subtypes, based on the histological appearance and characteristics of the tumor cells [7, 8, 9]. Chondroid chordoma is a type of conventional chordoma. The term “chondroid chordoma” refers to chordomas including a large area of extracellular matrix mimicking hyaline cartilaginous tumors [10]. The prognosis of chondroid chordomas is better than that of conventional chordomas [10, 11]. Even among conventional chordomas, there is heterogeneity in extracellular matrix (ECM) phenotypes. Furthermore, there is a variable prognosis among conventional chordomas.

Several proteases that digest extracellular matrix collagens, including matrix metalloproteinase (MMP) 1, MMP2, MMP9, cathepsin K, and cathepsin B, were produced in chordomas [12, 13, 14, 15]. However, one of the major collagenases, MMP13, has not been examined in chordomas.

In the present study, we categorized conventional chordomas by ECM characteristics using safranin‐O staining. We also quantified MMP13 expression in patient tissues to test its clinical association with ECM phenotype and MMP expression. To examine the function of MMP13, we used JHC7 chordoma cells to evaluate collagenolytic activity and the effect of a selective MMP13 inhibitor, and to compare cell growth in the presence or absence of collagens (COL1 and COL2).

## 2 Materials and Methods

### 2.1 Study Population

We retrospectively analyzed data from patients who underwent surgery for primary conventional chordoma of the skull base at our institute from 1985 to 2019. All tumor samples were obtained at initial surgery, prior to any radiation therapy or pharmacological therapy. No patient had recurrent disease at the time of sampling. All procedures performed in this study involving human subjects were carried out in accordance with the ethical standards of the Keio University School of Medicine Ethics Committee (Reference number: 20050002) and with the 1964 Helsinki Declaration and its later amendments or comparable ethical standards. Informed consent was obtained from all patients. Thirty‐seven primary skull base chordomas were analyzed (Table 1). All cases underwent safranin‐O staining, and 36 cases were traceable and evaluable in terms of progression‐free survival (PFS), defined as the interval between the date of first surgery and second surgery for recurrence. Additional clinical information, including tumor volume, comorbidities, medications potentially affecting gene expression, and the presence of intradural and cavernous sinus invasion, is summarized in Table S1. Because these data were collected retrospectively from electronic medical records, several data fields were unavailable and are indicated as “N/A” in Table S1.

**TABLE 1:** Patient's characteristics.

Clinical feature	
Patient number	37
Age (mean ± SD)	47 ± 20 (9–78)
Sex	
Male	19
Female	18
Histological appearance	
Safranin‐O negative chordoma	15
Safranin‐O positive chordoma	22

### 2.2 Antibodies

The following antibodies were used: goat anti‐brachyury (R&D Systems, AF2085, RRID:AB_2200235), mouse anti‐brachyury (Abcam, ab140661, RRID:AB_3076540), rabbit anti‐MMP13 (Abcam, ab39012, RRID:AB_776416), rabbit anti‐MMP9 (Abcam, ab38898, RRID:AB_776512), goat anti‐MMP9 (R&D Systems, AF909, RRID:AB_355706), mouse CD24 (BD Biosciences, 555426, RRID:AB_395820), and mouse anti‐cathepsin K (DFK, F‐95, RRID:AB_2927739).

### 2.3 Immunohistochemistry

Formalin‐fixed paraffin‐embedded sections (4 μm) of surgical specimens were used for histopathological analyses. Chordoma is an extremely rare disease, and due to the wide variation in the preservation periods of the samples used in this study, we first assessed the preservation of antigenicity for immunohistochemical analysis by evaluating the samples using brachyury, a well‐established marker of chordoma. Sections were deparaffinized and rehydrated. Antigen retrieval was performed in citrate buffer (10 mM, pH 6.0, 37°C, 30 min) for MMP13, cathepsin K, and brachyury and in 10 μg/mL protease K/20 mM Tris–HCl, pH 9.0 (room temperature, 10 min) for MMP9. Sections were blocked for 60 min in blocking solution [1.0% (wt/vol) bovine serum albumin (BAC62, Equitech‐Bio), 5.0% (vol/vol) normal donkey serum (D9663‐10 mL, Sigma‐Aldrich), and 10 μg/mL donkey IgG (017‐000‐003, Jackson ImmunoResearch Laboratories) in 0.02% Triton‐X100/phosphate‐buffered saline (PBS)]. The sections were then incubated overnight at 4°C with goat anti‐brachyury (5 μg/mL), mouse anti‐cathepsin K (5 μg/mL), rabbit anti‐MMP9 (2 μg/mL), or rabbit anti‐MMP13 antibodies (5 μg/mL), followed by anti‐goat, mouse, or rabbit IgG secondary antibody (ImmPRESS Detection Systems, Vectorlabs) for 60 min at room temperature. The products were visualized following the peroxidase‐diaminobenzidine reaction. For immunofluorescent staining, antigen retrieval was performed in 10 μg/mL protease K/20 mM Tris–HCl, pH 9.0 (room temperature, 10 min) and mouse anti‐brachyury (2 μg/mL), goat anti‐MMP9 (5 μg/mL), and rabbit anti‐MMP13 antibodies (4 μg/mL) were used. Nuclei were stained with diamidino‐2‐phenylindole (DAPI, Sigma‐Aldrich), and Alexa plus 488/555/647‐conjugated secondary antibodies (Thermo Fisher Scientific), TrueVIEW Autofluorescence Quenching Kit (SP‐8400, Vector Laboratories), and ProLong Glass Antifade Mountant (P36980, Thermo Fisher Scientific) were used. Sections were observed under a confocal laser scanning microscope (FV3000, Evident) or with a virtual slide scanner (NanoZoomer, Hamamatsu Photonics).

### 2.4 Safranin‐O Staining

Paraffin sections were stained with safranin‐O staining by sequential soaking in Weigert's iron hematoxylin solution (10 min), 0.5% hydrochloric acid in ethanol (6 times), running tap water (10 min), 0.05% Fast Green solution (Polysciences, Warrington) (5 min), 1% acetic acid solution (10 s), and 0.1% safranin‐O solution (Polysciences) (5 min). The sections were then dehydrated and cleared with 95% ethyl alcohol and isopropanol, and mounted using VectaMount Express (H‐5700‐60, Vector Laboratories).

### 2.5 MMP13 Scoring

The intensity of MMP13 staining in five brachyury‐positive fields in each human chordoma sample was evaluated by five independent investigators, using a blinded method, at 10× magnification using a virtual slide image (NanoZoomer, Hamamatsu Photonics). Sections were assigned scores from 1 (no staining) to 5 (intensely stained). For two exceptionally small samples, three fields were evaluated.

### 2.6 Cells

JHC7 cells were obtained from ATCC (CRL‐3267, Lot: 63327609, RRID:CVCL_L154) and cultured in Dulbecco's Modified Eagle Medium (DMEM)/Ham's F‐12 medium (Nacalai, 08460‐95) supplemented with 10% fetal bovine serum and an antibiotic‐antimycotic reagent (Thermo Fisher, 15240062). HEK293FT, U‐87 MG, and HeLa cells were obtained from Thermo Fisher Scientific (R70007, RRID:CVCL_6911), ATCC (HTB‐14, RRID:CVCL_0022), and RIKEN Bioresource Center (RCB0007, RRID:CVCL_0030), respectively, and cultured in DMEM high‐glucose medium (Nacalai, 0845845), MEM α, nucleosides (Thermo Fisher, 12571063), or DMEM low‐glucose medium (Nacalai, 08456‐65) supplemented with 10% fetal bovine serum and an antibiotic‐antimycotic reagent (Thermo Fisher, 15240062).

### 2.7 Immunocytochemistry

JHC7 cells were cultured on glass coverslips (Matsunami, C018001) coated with 0.1% gelatin (Sigma‐Aldrich, G1393) for 7 days, fixed with 4.0% paraformaldehyde in PBS for 10 min, permeabilized with 0.1% Triton X‐100 in PBS for 5 min, blocked with 1.0% BSA with 5.0% donkey serum (Sigma‐Aldrich, D9663) and 10 μg/mL normal donkey IgG (Jackson ImmunoResearch Laboratories, 017‐000‐003) in PBS for 60 min. The cells were then stained with the indicated primary antibodies (2 μg/mL in 1% BSA/PBS) overnight at 4°C, followed by three washes with PBS (15 min total). The appropriate secondary antibodies [Thermo Fisher, Alexa Fluor plus 555‐conjugated donkey anti‐rabbit IgG (A32794), Alexa Fluor Plus 488‐conjugated donkey anti‐mouse IgG (A32766), or Alexa Fluor 647‐conjugated donkey anti‐goat IgG (A‐21447)] were applied with DAPI (Sigma‐Aldrich, D9542) for 60 min at room temperature. After washing with PBS, the coverslips were mounted with ProLong Diamond Antifade Mountant (Thermo Fisher, P36961) and observed under a confocal laser scanning microscope (FV3000, Evident).

### 2.8 Real‐Time Quantitative Polymerase Chain Reaction (PCR) Analysis

Total RNA was prepared from cultured cells using a FastGene RNA Basic Kit (Nippon Genetics, FG‐80006) and reverse‐transcribed using ReverTra Ace qPCR RT Master Mix with gDNA Remover (Toyobo, FSQ‐301). Expression levels were quantified by fluorescence‐based real‐time PCR using a ViiA7 Real‐Time PCR System (Thermo Fisher) with THUNDERBIRD SYBR qPCR (Toyobo, QPS‐201) and were normalized to the glyceraldehyde 3‐phosphate dehydrogenase gene (*GAPDH*) as an internal standard. The primer sequences are shown in Table 2. The PCR was performed as follows: pre‐denaturation at 95°C for 20 s and 45 cycles (95°C for 1 s, 63°C for 20 s).

**TABLE 2:** Primer sequencing.

Target gene	Sense primer	Tm[W]^a^ (°C)	Tm[V]^b^ (°C)	Antisense primer	Tm[W]^a^ (°C)	Tm[V]^b^ (°C)
*GAPDH*	GAAGGTGGTGAAGCAGGCGTC	68	70.29	ATGCCAGCCCCAGCGTCAAAG	68	74.3
*TBXT*	ATTTGACTGCTCTGCCCCCTAG	68	67.4	GCTTTCTGCTGATTGTCTTTGGC	68	67.91
*MMP1*	GGATTCATATAGGCCAGAGTTGC	68	64.49	TGTTTGTCACTGAAGCTGCTCTC	68	65.74
*MMP2*	CAGGGCACAGGTGATGGTGTC	68	70.2	TTTCTACAGGACAGAGGGACTAG	68	60.31
*MMP9*	GTGCCATGTAAATCCCCACTGG	68	69.18	ACTCCTCCCTTTCCTCCAGAAC	68	65.69
*MMP13*	ATATGACTATGCGTGGCTGGAAC	68	65.84	GTGGTGTGGGAAGTATCATCAAC	68	64.17
*CTSK*	CCAAGATGTGACTCCAGCCAGC	70	70.27	AAATAGCACACCAACTCCCTTCC	68	66.02

^a^ Wallace method [2(A + T) + 4(G + C)].

^b^ van't Hoff equation (CONC. 50 mM‐salt, 0.5 μM‐primer).

### 2.9 Collagen Digestion Assay in Live Cells

Fluorescein isothiocyanate‐labeled bovine type II collagen (FITC‐COL2, 4002, Chondrex) was mixed with Matrigel (growth factor‐reduced, phenol‐red free, 356231, Corning) in a 1:1 ratio and placed on a polymer coverslip in the bottom of a 96‐well plate (89626, Ibidi). The coated plate was washed three times with PBS and once with culture medium. JHC7 cells (2 × 10^5^ cells/well) were then cultured on the FITC‐COL2 coated plate. Culture supernatant was collected when the medium was changed (once every 3–4 days) and mixed with 1 M Tris–HCl (pH 9.0, 10% volume of supernatant) to achieve an optimal pH for fluorescence measurement.

Released FITC in the supernatant was measured using a microplate reader (Citation 5, Agilent). The remaining FITC‐COL2 on the plate was also measured from the bottom side using a microplate reader (SpectraMax, Molecular Devices). Culture supernatant without FITC‐COL2 coating was used as background and culture medium without JHC7 cells was used as a negative control.

Released FITC and remaining FITC‐COL2 were measured in the presence of JHC7 cells treated with the MMP13‐specific inhibitor, CAS 544678‐85‐5 [16], and dimethylsulfoxide (DMSO) was used as a negative control.

### 2.10 Cell Proliferation Assay

JHC7 cells were cultured on a 96‐well plate for 24 h. The culture medium was then replaced with culture medium supplemented with various concentrations of type I or type II atelocollagen (IPC‐30 or CL‐22, KOKEN) dissolved in 1 mM HCl. An equivalent amount of HCl was used as control. Imaging was performed using an IncuCyte S3 live‐cell analysis system (Sartorius). The cell‐growth rate was calculated by linear regression.

### 2.11 Statistical Analysis

MMP13 scores were compared between safranin‐O negative and positive chordomas by Student's *t*‐test. PFS was estimated using the Kaplan–Meier method. The resultant Kaplan–Meier curves crossed and violated the proportional hazard assumptions underlying the log rank test and Cox regression model. We therefore used the restricted mean survival time (RMST) method to compare PFS between patients with safranin‐O negative and positive chordomas during a 150‐month period. PFS analyses were performed using R (version 4.2.1). Collagen digestion assays were compared using Student's *t‐*test. Cell proliferation assays were analyzed by ANOVA and Dunnett's test, using IgorPro 9.0 software (HULINKS). A *p* value < 0.05 was considered statistically significant.

## 3 Results

### 3.1 Safranin‐O Staining and PFS in Patients With Chordomas

**FIGURE 1:**
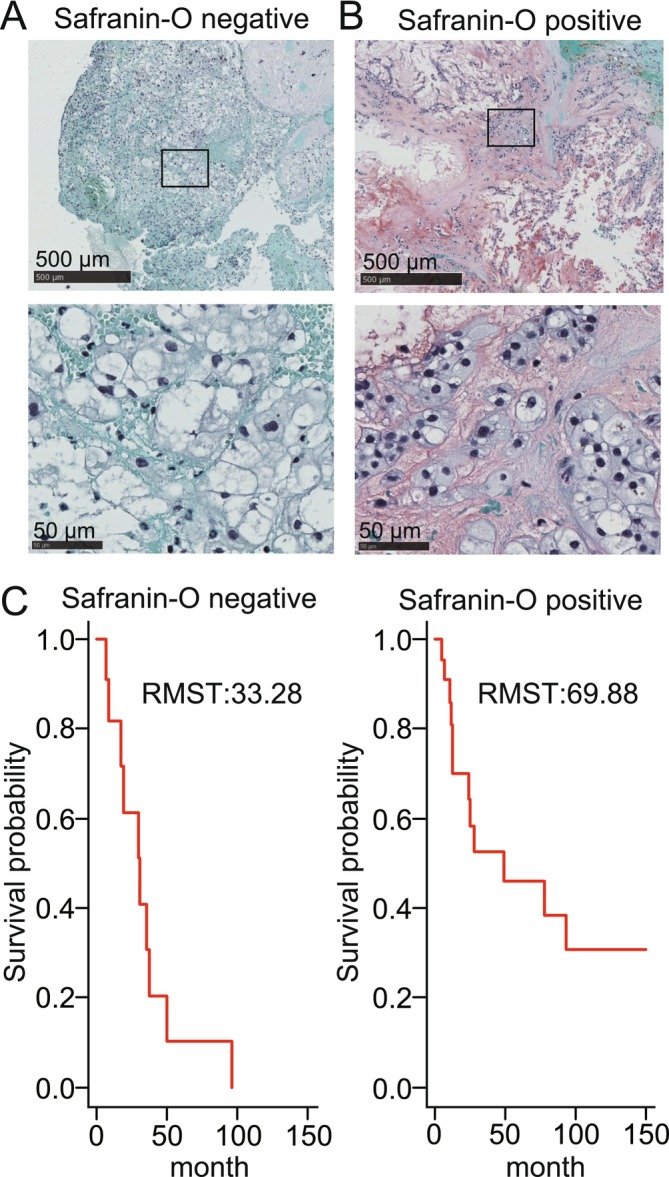
Classification by safranin‐O staining and progression‐free survival analysis. (A, B) Safranin‐O staining (red) of the cartilaginous extracellular matrix in safranin‐O negative chordoma (A) and safranin‐O positive chordoma (B), counterstained with fast green/iron‐hematoxylin. (C) Kaplan–Meier curves for progression‐free survival in safranin‐O negative chordoma and safranin‐O positive chordoma. RMST, restricted mean survival time.

We first applied safranin‐O/fast green staining to surgical chordoma specimens to visualize cartilage‐like, proteoglycan‐rich extracellular matrix (safranin‐O, red) against non‐cartilaginous components (fast green, green), and on this basis stratified all 37 conventional chordomas into safranin‐O‐positive (cartilage‐like ECM present) and safranin‐O‐negative groups. Fifteen cases were safranin‐O‐negative, composed of lobulated sheets of physaliphorous tumor cells separated by fibrous septa without detectable safranin‐O signal (Figure 1A), whereas the remaining 22 were safranin‐O‐positive, showing partial to diffuse safranin‐O reactivity within the cartilage matrix (Figure 1B). We compared the clinical features of safranin‐O negative and positive chordomas in 37 patients (19 male, 18 female) for whom clinical information and pathological specimens were available. The patient characteristics are summarized in Table 1. The age range of the study population was 9–78 years (mean 47 ± 20 years). The RMST differed significantly between safranin‐O positive and negative chordomas (33.23 months vs. 69.88 months, *p* = 0.016), indicating shorter PFS in the safranin‐O negative chordoma group (Figure 1C).

### 3.2 Collagenase Expression in Chordomas and MMP13 Expression Scores in Safranin‐O Negative and Positive Chordomas

**FIGURE 2:**
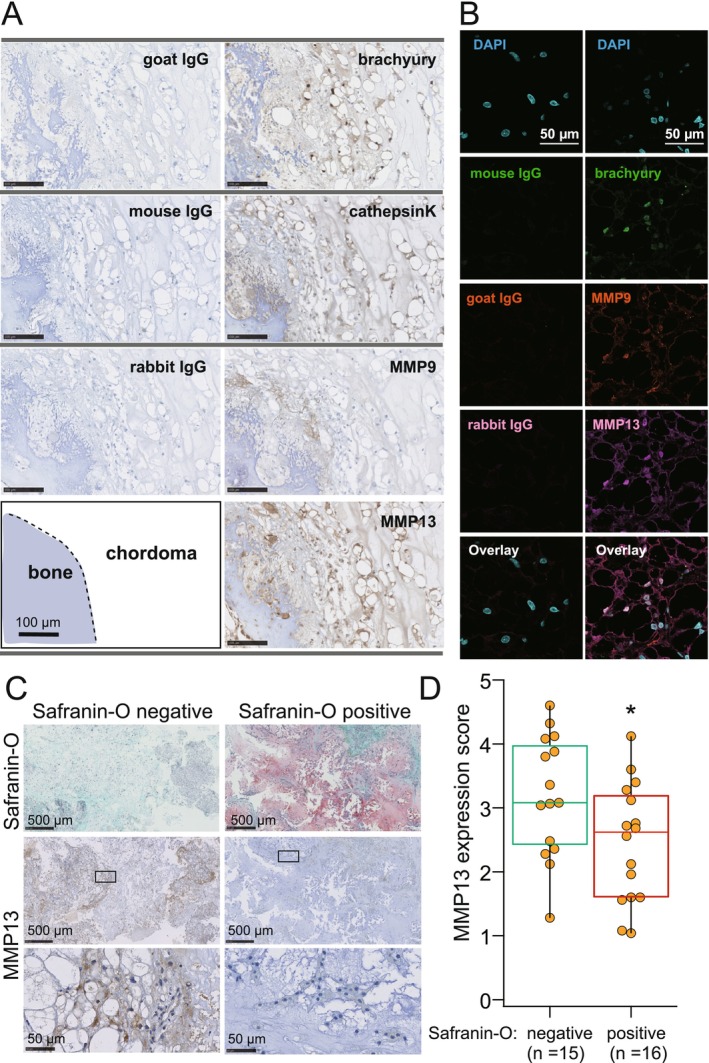
Expression and quantitative analysis of MMP13 in human skull base chordoma. (A) Immunohistochemical staining of brachyury, cathepsin K, MMP9, and MMP13 in a primary skull base chordoma with bone invasion. (B) Immunofluorescence showing coexpression of brachyury, MMP9, and MMP13 in the chordoma tissue shown in (A). (C) Representative MMP13 expression in safranin‐O negative and positive chordomas. (D) MMP13 expression scores of safranin‐O negative and positive chordomas. *p < 0.05.

We examined the possible factors influencing the prognosis of chordomas, focusing on the cartilaginous matrix. We therefore investigated expression levels of collagenases in chordomas. As reported previously [12, 13, 14, 15], cathepsin K and MMP9 were expressed in brachyury‐positive chordoma tissues (Figure 2A). As detected by immunofluorescence, MMP13 expression was also detected in chordomas, and brachyury, MMP9, and MMP13 were co‐expressed in human chordomas (Figure 2B).

Because the intensity of MMP13 expression in chordomas varied (Figure 2C), we quantified MMP13 expression levels to score its expression in safranin‐O negative and positive chordomas. To evaluate the MMP13 expression score, we used brachyury‐positive samples, as 31 of the 37 specimens showed the preservation of antigenicity for immunohistochemical analysis. The MMP13 expression score was significantly higher in safranin‐O negative chordomas compared with safranin‐O positive chordomas (Figure 2D *p* = 0.018).

### 3.3 Expression of Collagenases in JHC7 Chordoma Cells

**FIGURE 3:**
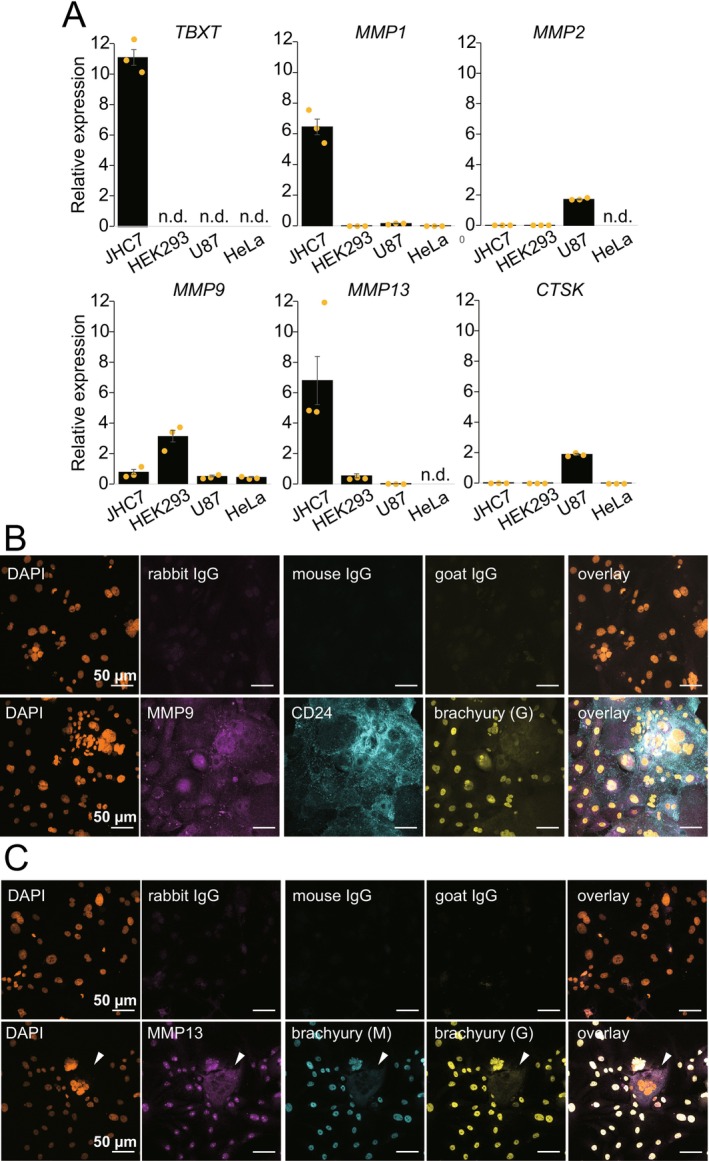
Expression of collagenases in JHC7 cells. (A) RNA expression levels of *TBXT, MMP1, MMP2, MMP9*, *MMP13*, *and CTSK* measured by quantitative real‐time polymerase chain reaction. HEK293, U‐87 MG (U87), and HeLa cells served as controls. Values were normalized to *GAPDH* expression. (B) Immunocytochemical staining of MMP9, CD24, and brachyury. (C) Immunocytochemical staining of MMP13 and brachyury. Note that two independent anti‐brachyury antibodies were used: (M) mouse anti‐brachyury antibody, (G) goat anti‐brachyury antibody. Arrowheads indicate cytosolic staining of MMP13 and brachyury in a giant JHC7 cell.

To examine the expression and function of collagenases in chordoma cells, we first analyzed the gene expression of collagenases and cathepsin K in JHC7 chordoma cells. JHC7 cells expressed *TBXT* (brachyury), *MMP1, MMP2, MMP9*, and cathepsin K (*CTSK*), consistent with previous reports showing that chordomas expressed MMPs and cathepsin K [12, 13, 14, 15]. In addition, MMP13 was highly expressed in JHC7 cells (Figure 3A). Consistent with chordoma tissues in Figure 2B, immunocytochemical analysis confirmed the expression of MMP9 (Figure 3B) and MMP13 (Figure 3C) in JHC7 cells. MMP9 immunostaining was observed in the cytosol of mono‐ or multinucleated JHC7 cells expressing the chordoma nuclear marker brachyury and the chordoma cell surface marker CD24 (Figure 3B). MMP13 expression was predominantly detected in the nucleus in mono‐ or multinucleated JHC7 cells (Figure 3C). In mononuclear JHC7 cells, both MMP13 and brachyury were localized in the nucleus. By contrast, in the occasionally observed multinucleated giant cells, both MMP13 and brachyury [17] were localized in the cytoplasm (Figure 3C, arrowhead).

### 3.4 COL2 Digestion Assay in JHC7 Cells

**FIGURE 4:**
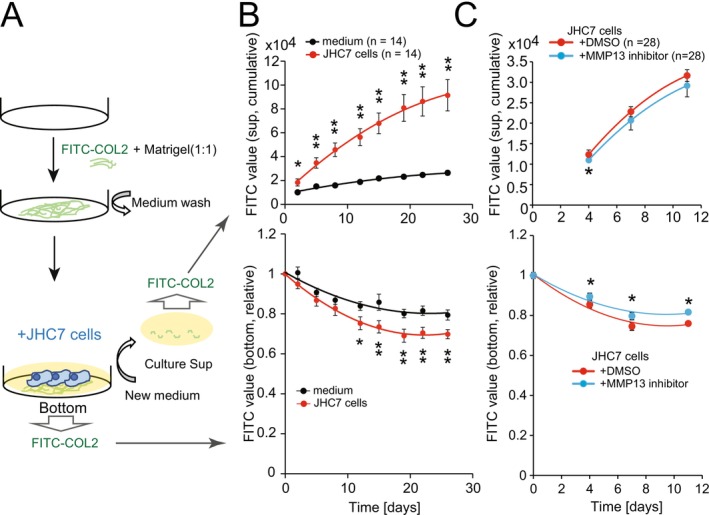
Digestion assay of type II collagen (COL2) by JHC7 cells. (A) Schematic of collagen digestion assay using JHC7 cells cultured on a FITC‐COL2 fluorescent plate. (B) After culturing JHC7 cells for the indicated days, fluorescence intensities of FITC‐COL2 were measured in both the culture supernatant (upper) and on the plate (lower). (C) Digestion of FITC‐COL2 by JHC7 cells in the presence or absence of MMP13 inhibitor. COL2, collagen; DMSO, dimethylsulfoxide; FITC, fluorescein isothiocyanate. *p < 0.05, **p < 0.01.

COL2 is the major substrate of MMP13 and an abundant component of the cartilaginous matrix [18, 19]. We therefore analyzed the collagenase activity in JHC7 cells using culture plates coated with ECM containing FITC‐COL2 (Figure 4A). FITC release in the supernatant and decreased attachment of FITC‐COL2 were significantly enhanced in the presence of JHC7 cells (Figure 4B). We assessed endogenous MMP13 activity in JHC7 cells using the MMP13‐specific inhibitor, CAS 544678‐85‐5 [16]. Enhancement of FITC release by JHC7 cells was partially but significantly inhibited by the MMP13‐specific inhibitor (Figure 4C). These results suggest that MMP13 secreted by JHC7 cells is responsible for the degradation of COL2 in the ECM.

### 3.5 Growth Inhibition of JHC7 Cells by Collagens

**FIGURE 5:**
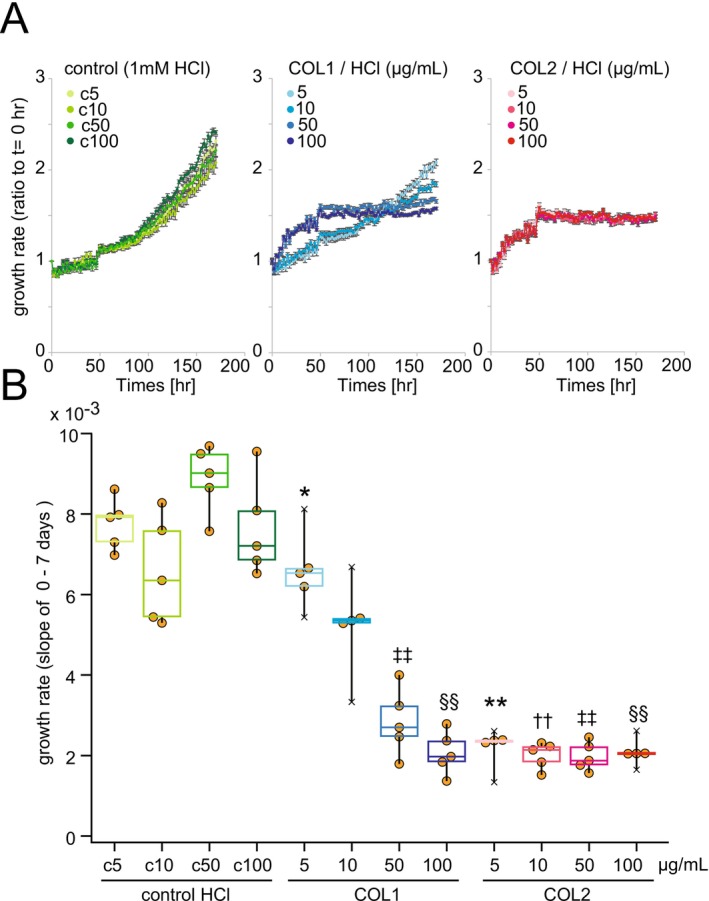
Growth inhibition of JHC7 by collagens. (A) Growth curves of JHC7 cells in the presence or absence of collagens. (B) Quantitation of growth rate of JHC7 cells in (A). Individual data plotted as circles; crosses indicate outliers. c5, c10, c50, and c100 indicate 1.65, 3.3, 16.5, and 33 μL 1 mM HCl in 1 mL medium, equivalent to 5, 10, 50, and 100 μg/mL collagens in 1 mM HCl, respectively. *N* = 5, *p < 0.05, **p < 0.01, compared with c5, ^††^ *p* < 0.05 compared with c10, ^‡‡^ *p* < 0.05 compared with c50, ^§§^ *p* < 0.01 compared with c100 (Dunnett's test).

Finally, we assessed the effect of collagen on the proliferation of JHC7 cells, to investigate the relationship between the ECM and tumor progression (Figure 5). The growth rate of JHC7 cells in culture medium with or without type I collagen (COL1) or COL2 was monitored using a live‐cell analysis system. COL1 inhibited cell growth in a dose‐dependent manner, and COL2 inhibited cell growth more strongly than COL1, even at low concentrations.

These findings suggest that MMP13 digests inhibitory COL2 (cartilaginous matrix) and may thus help to promote the aggressive growth of safranin‐O negative chordomas.

## 4 Discussion

Chordomas cause bone destruction and local invasion into the surrounding area and express a variety of proteases (cathepsin K, cathepsin B, MMP1, MMP2, and MMP9) [12, 13, 14, 15]. MMPs in turn promote tumor invasion by decomposing ECM proteins [20]. MMP13 is a collagenase that cleaves COL2 (the major component of cartilage) five times faster than COL1 (the major component of the bone matrix), six times faster than collagen III, and more actively than other collagenases [21, 22]. MMP13 has also been shown to promote tumor progression in a variety of human malignancies [23], however, information on MMP13 expression in chordomas is lacking. We therefore investigated the expression of MMP13 in human chordoma specimens and JHC7 cells, and found that the MMP13 expression score was significantly higher in safranin‐O negative chordomas compared with safranin‐O positive chordomas. In addition, PFS was shorter in patients with safranin‐O negative chordomas than in patients with safranin‐O positive chordomas. This finding is consistent with previous studies indicating that patients with chondroid chordoma show a longer PFS [10, 11]. These results suggest that MMP13 degrades the cartilaginous (chondroid) ECM and appears to be an unfavorable prognostic factor in patients with chordomas.

Chordomas are characterized by the abnormal production of ECM components, which also contribute to their histological identification [24]. The expression of COL2, which is a typical marker of chondrocytic differentiation [25], has been reported in chordomas [26, 27], and the current study also detected hyaline cartilage in chordoma specimens by safranin‐O staining. COL2 is essential for the development of the notochord into the nucleus pulposus [28], with notochord cells becoming part of the nucleus pulposus and starting to express COL2 [26]. In chondrosarcomas, COL2, which is characteristic of differentiated chondrocytes, indicates a mature neoplastic cell phenotype associated with good prognosis [29]. COL2, which indicates a more differentiated state of the notochord, may thus be a favorable prognostic factor in patients with chordomas.

As noted above, COL2 expression in chordomas is thought to be important in terms of tumor differentiation and clinical outcomes. We therefore focused on the expression of MMP13 in chordomas, given that it degrades COL2 more actively than other collagenases. To situate our findings within the broader cartilage‐tumor context, we briefly reference malignant cartilage tumors (chondrosarcomas) as a mechanistic comparator, not as a disease equivalent to chordoma. Although MMP13 expression has not been reported in chordomas, it has been observed in chondrosarcomas [30, 31, 32, 33], which share similarities with chordomas in terms of a cartilaginous matrix around the tumor cells and destruction of the surrounding bone [34]. Grade II and III chondrosarcomas, which have less chondroid matrix and more mitotic activity than grade I chondrosarcomas [35], showed significantly higher expression of MMP13 [30, 32]. These results suggest that MMP13 expression may correlate with degradation of the chondroid matrix and histological malignancy in chondrosarcomas, as seen in chordomas in the present study.

In addition to its cytoplasmic expression, we observed that MMP13 was expressed in the nucleus of tumor cells in chordoma specimens and was predominantly expressed in the nucleus of JHC7 cells. MMPs are normally localized in cytosol, organelles, and extracellular compartments, but many types of MMPs have recently been found in nucleus [36]. MMPs contain a nuclear localization signaling sequence that allows them to enter the nucleus and regulate certain nuclear events [37, 38]. Nuclear MMP13 has been found in neural cells after cerebral ischemia, suggesting that its nuclear role is crucial in the ischemia‐induced apoptotic cascade [39]. Xie et al. proposed that cells that can survive the natural selection by apoptosis induced by nuclear MMPs may have undergone a second gene mutation, abnormal signaling elevation, or cross‐talk signaling complementation, which might allow them to survive and promote more aggressive diseases [36]. Although nuclear MMPs may be associated with aggressive cancer progression [40], the function of nuclear MMP13 in chordoma is unknown and further studies are warranted.

The current results also showed that JHC7 cells expressed MMP13 and had collagenolytic activity, and the COL2‐digestion activity of JHC7 cells was suppressed by an MMP13‐specific inhibitor. Notably, the JHC7 cell‐growth rate was strongly inhibited in the presence of COL2 in a cell‐based assay. These data suggest that COL2 may inhibit tumor growth in chordomas, and we therefore hypothesized that increased MMP13 expression in safranin‐O positive chordomas, which are rich in growth‐inhibitory COL2, may contribute to tumor progression through collagen degradation, and MMP13 and COL2 may thus be therapeutic targets in chordomas. The ECM can form a physical barrier that prevents tumor cell proliferation and invasion [41]. Within the ECM, tissue inhibitors of metalloproteinases (TIMPs) inhibit the proteolytic activity of MMPs, and an imbalance between MMPs and TIMPs has been implicated in tumor progression [42]. Although the expression of TIMPs in chordoma has been confirmed [14], their clinical significance remains unclear. Recent studies demonstrated that recombinant TIMPs or overexpression of TIMPs inhibited cancer cell invasion in vitro and in vivo [43]. Additionally, periosteum thickening (COL1) promoted by TIMP1 has been shown to protect against cancer cell invasion into bone [44]. The transplantation of COL2 materials, such as gels, or the administration of TIMP1 into the periosteum after chordoma removal surgery may be able to suppress tumor growth and invasion.

This study had some limitations. The sample size was small because of the rarity of chordomas (0.08 in a million per year) [4]. Further studies with more patients are therefore warranted to confirm the findings of this study. In addition, extending the scope beyond clival chordoma to include observations of spinal and sacral chordomas may allow generalization of the observation that chordomas are exacerbated by the digestion of growth‐inhibitory collagens.

## 5 Conclusion

MMP13 was expressed in chordoma tissues and JHC7 cells. Safranin‐O‐negative chordomas exhibit higher levels of MMP13 and shorter PFS. JHC7 cells digest COL2 in an MMP13‐dependent manner, and COL2 suppresses cell growth. Together, these findings support a model in which MMP13 degrades COL2‐rich, growth‐inhibitory ECM, facilitating aggressive behavior. Future studies should validate MMP13 as a prognostic marker in larger, multicenter cohorts (including spinal and sacral chordomas) and evaluate the efficacy of selective MMP13 inhibition or ECM‐stabilizing strategies (e.g., TIMPs or COL2‐based materials), in preclinical chordoma models.

## Author Contributions

**Yumiko Oishi:** data curation, investigation, visualization, writing – review and editing, writing – original draft, formal analysis, resources. **Katsuhiro Kawaai:** conceptualization, methodology, data curation, investigation, formal analysis, supervision, funding acquisition, visualization, writing – original draft, writing – review and editing. **Ryota Tamura:** conceptualization, writing – review and editing, resources, data curation. **Yukiko Kuroda:** conceptualization, formal analysis, visualization, writing – review and editing, supervision, investigation. **Shinobu Noji:** validation, formal analysis, writing – review and editing. **Masahiro Toda:** conceptualization, resources, supervision, writing – review and editing, project administration. **Koichi Matsuo:** conceptualization; writing – original draft; writing – review and editing, supervision, visualization, project administration.

## Funding

This research was supported by JSPS KAKENHI (Grant 22K09217) (KK) and a grant‐in‐aid from Keio University Sakaguchi‐Memorial Medical Science Fund (KK).

## Ethics Statement

All procedures performed involving human subjects were in accordance with the ethical standards of the Keio University School of Medicine Ethics Committee (approval number: 20050002) and with the 1964 Helsinki Declaration and its later amendments or comparable ethical standards. Informed consent was obtained from all patients.

## Consent

Informed consent was obtained from all individual participants included in the study.

## Conflicts of Interest

The authors declare no conflicts of interest.

## Supporting information


**Table S1:** Clinical characteristics of patients with conventional chordoma. N/A indicates data not available due to limitations of electronic medical record retrieval; “none” indicates explicit documentation of absence. Medications affecting gene expression (steroids, anticancer agents, and hormonal drugs) were recorded if used preoperatively. Intradural and cavernous sinus invasion are indicated as “+” (present) or “−” (absent) based on preoperative imaging review and/or intraoperative findings. PFS (progression‐free survival) is defined as the interval (months) between the first surgery and the second surgery for tumor recurrence. Patients without reoperation for recurrence at the time of last clinical follow‐up were considered censored; these cases are marked with “+” in the PFS column.

## Data Availability

The datasets analyzed in this study are available from the corresponding author on reasonable request.
