# Supplementary material for: Matrix Metalloproteinase‐13 Is an Unfavorable Prognostic Factor in Chordoma by Digesting Growth Inhibitory Collagens
Source: Cancer Med. 2026 Feb 19;15(2):e71662. doi: 10.1002/cam4.71662 (PMC12920066; doi:10.1002/cam4.71662)
Supplement: Supplementary file 1 — Table S1: Clinical characteristics of patients with conventional chordoma. N/A indicates data not available due to limitations of electronic medical record retrieval; “none” indicates explicit documentation of absence. Medications affecting gene expression (steroids, anticancer agents, and hormonal drugs) were recorded if used preoperatively. Intradural and cavernous sinus invasion are indicated as “+” (present) or “−” (absent) based on preoperative imaging review and/or intraoperative findings. PFS (progression‐free survival) is defined as the interval (months) between the first surgery and the second surgery for tumor recurrence. Patients without reoperation for recurrence at the time of last clinical follow‐up were considered censored; these cases are marked with “+” in the PFS column. [file CAM4-15-e71662-s001.docx]

Table S1 : Clinical characteristics of patients with conventional
chordoma. N/A indicates data not available due to limitations of

electronic medical record retrieval; 'none' indicates explicit

documentation of absence. Medications affecting gene expression

(steroids, anticancer agents, and hormonal drugs) were recorded if used

preoperatively. Intradural and cavernous sinus invasion are indicated as

“+” (present) or “−” (absent) based on preoperative imaging review

and/or intraoperative findings. PFS (progression-free survival) is

defined as the interval (months) between the first surgery and the

second surgery for tumor recurrence. Patients without reoperation for

recurrence at the time of last clinical follow-up were considered

censored ; these cases are marked with “+” in the PFS column.

| Case | Age | Sex | Pathological diagnosis | Safranin-O staining | Tumor volume (cm^3^) | Comorbidities | Medications affecting gene expression | Intradural invasion | Cavernous sinus invasion | PFS (months) |
| --- | --- | --- | --- | --- | --- | --- | --- | --- | --- | --- |
| 1 | 14 | M | conventional chordoma | + | N/A | N/A | N/A | + | - | 174+ |
| 2 | 53 | M | conventional chordoma | + | N/A | N/A | N/A | + | N/A | 28 |
| 3 | 62 | M | conventional chordoma | - | 12.7 | N/A | N/A | - | - | N/A |
| 4 | 57 | F | conventional chordoma | + | 44 | N/A | N/A | + | + | 49 |
| 5 | 25 | F | conventional chordoma | + | N/A | N/A | N/A | - | + | 11 |
| 6 | 10 | F | conventional chordoma | + | 22.3 | N/A | N/A | - | - | 78 |
| 7 | 60 | M | conventional chordoma | + | N/A | N/A | N/A | + | - | 12 |
| 8 | 53 | F | conventional chordoma | + | N/A | N/A | N/A | + | - | 25 |
| 9 | 9 | F | conventional chordoma | - | N/A | N/A | N/A | + | - | 29 |
| 10 | 57 | F | conventional chordoma | + | N/A | N/A | N/A | - | - | 13 |
| 11 | 44 | F | conventional chordoma | + | N/A | N/A | N/A | + | + | 8+ |
| 12 | 70 | F | conventional chordoma | + | N/A | N/A | N/A | + | N/A | 7 |
| 13 | 30 | M | conventional chordoma | + | N/A | N/A | N/A | - | - | 164+ |
| 14 | 12 | F | conventional chordoma | - | N/A | N/A | N/A | + | + | 17 |
| 15 | 59 | M | conventional chordoma | + | N/A | N/A | N/A | + | + | 112+ |
| 16 | 28 | M | conventional chordoma | - | 8.8 | N/A | N/A | - | + | 37 |
| 17 | 31 | M | conventional chordoma | + | 24.2 | none | none | - | + | 117+ |
| 18 | 43 | M | conventional chordoma | + | 28.8 | none | none | + | - | 5 |
| 19 | 62 | F | conventional chordoma | - | 7.8 | none | none | - | + | 8 |
| 20 | 58 | F | conventional chordoma | - | 23 | none | none | - | - | 19 |
| 21 | 78 | F | conventional chordoma | + | 177.5 | Cataract, subarachnoid hemorrhage | none | + | + | 13 |
| 22 | 74 | M | conventional chordoma | + | 10.4 | Colorectal cancer | none | - | - | 65+ |
| 23 | 49 | M | conventional chordoma | + | N/A | N/A | N/A | N/A | N/A | 24 |
| 24 | 32 | M | conventional chordoma | + | 13.1 | none | none | + | - | 32+ |
| 25 | 74 | F | conventional chordoma | - | 6.6 | Cataract, hyperlipidemia, hypertension, and ovarian cyst | Hydrocortisone 15 mg/day | + | + | 2+ |
| 26 | 48 | M | conventional chordoma | - | 17 | none | none | - | - | 4+ |
| 27 | 70 | M | conventional chordoma | + | 11.1 | none | none | + | + | 12+ |
| 28 | 77 | M | conventional chordoma | - | 21 | Diabetes mellitus, sudden sensorineural hearing loss, and hyperlipidemia | none | + | + | 8+ |
| 29 | 68 | M | conventional chordoma | - | 17.5 | none | none | - | + | 50 |
| 30 | 46 | F | conventional chordoma | - | N/A | N/A | N/A | + | N/A | 96 |
| 31 | 71 | M | conventional chordoma | + | N/A | N/A | N/A | N/A | N/A | 93 |
| 32 | 45 | M | conventional chordoma | - | 6.9 | Hypertension (primary aldosteronism), depression, and nasal polyps | none | + | - | 6 |
| 33 | 25 | F | conventional chordoma | + | N/A | N/A | N/A | + | - | 10+ |
| 34 | 34 | F | conventional chordoma | - | N/A | N/A | N/A | + | + | 35 |
| 35 | 19 | F | conventional chordoma | + | 19.2 | none | none | + | - | 15+ |
| 36 | 41 | F | conventional chordoma | - | 25.5 | none | none | - | - | 30 |
| 37 | 41 | M | conventional chordoma | - | 20.2 | pure germinoma | none | + | + | 1+ |

N/A indicates data not available due to limitations of electronic medical record retrieval; 'none' indicates explicit documentation of absence.

Medications affecting gene expression: steroids, anticancer agents, and hormonal drugs were recorded if used preoperatively.

Intradural invasion and Cavernous sinus invasion are indicated as “+” (present) or “−” (absent), based on preoperative imaging review and/or intraoperative findings.

PFS (months): the interval between the first surgery and the second surgery for tumor recurrence. Patients without reoperation for recurrence at the time of last clinical follow-up were considered censored; these cases are marked with '+'.
